# A simple model for electrical charge in globular macromolecules and linear polyelectrolytes in solution

**DOI:** 10.1063/1.4983485

**Published:** 2017-05-24

**Authors:** M. Krishnan

**Affiliations:** Department of Chemistry, University of Zurich, Winterthurerstrasse 190, CH 8057 Zurich, Switzerland and Department of Physics, University of Zurich, Winterthurerstrasse 190, CH 8057 Zurich, Switzerland

## Abstract

We present a model for calculating the net and effective electrical charge of globular macromolecules and linear polyelectrolytes such as proteins and DNA, given the concentration of monovalent salt and pH in solution. The calculation is based on a numerical solution of the non-linear Poisson-Boltzmann equation using a finite element discretized continuum approach. The model simultaneously addresses the phenomena of charge regulation and renormalization, both of which underpin the electrostatics of biomolecules in solution. We show that while charge regulation addresses the true electrical charge of a molecule arising from the acid-base equilibria of its ionizable groups, charge renormalization finds relevance in the context of a molecule’s interaction with another charged entity. Writing this electrostatic interaction free energy in terms of a local electrical potential, we obtain an “interaction charge” for the molecule which we demonstrate agrees closely with the “effective charge” discussed in charge renormalization and counterion-condensation theories. The predictions of this model agree well with direct high-precision measurements of effective electrical charge of polyelectrolytes such as nucleic acids and disordered proteins in solution, without tunable parameters. Including the effective interior dielectric constant for compactly folded molecules as a tunable parameter, the model captures measurements of effective charge as well as published trends of $\text{p}K_{\text{a}}$ shifts in globular proteins. Our results suggest a straightforward general framework to model electrostatics in biomolecules in solution. In offering a platform that directly links theory and experiment, these calculations could foster a systematic understanding of the interrelationship between molecular 3D structure and conformation, electrical charge and electrostatic interactions in solution. The model could find particular relevance in situations where molecular crystal structures are not available or rapid, reliable predictions are desired.

## I. INTRODUCTION

The electrostatic properties of macromolecules—specifically, their electrical charge and interior dielectric characteristics—are a vital component of their function, contributing to the physical basis of mechanisms ranging from molecular recognition, signaling, and enzymatic catalysis, to protein folding and aggregation, and are of fundamental relevance in experiment and theory.^1–4^ “Supercharged” isoforms of evolutionarily conserved proteins are known to confer extreme physiological capacities on certain species, presumably owing to their enhanced stability to aggregation at high concentration.^5,6^ It is also well known that the addition and removal of small amounts of structural charge in the form of phosphate groups or other post-translational modifications modulates not only such basic phenomena as protein stability but also sub-cellular localization or function and can regulate macroscopic processes such as metabolism at the systemic level.^4^ Given the dominant role of an electrical charge in macromolecular interactions and function, theoretical models capable of predicting molecular electrical properties, e.g., effective electrical charge in solution, interior dielectric function and interaction free energies under arbitrary conditions, and making a direct connection to experiments are of great interest.

Contrary to the situation in vacuum, the electrical charge of a macromolecule in solution is governed strongly by thermodynamic processes in the electrolyte that render both theoretical predictions and experimental measurements of the quantity non-trivial. At the simplest level, a direct sum over a macromolecule’s charged groups yields a qualitative estimate of its formal structural electrical charge, at a given solution pH

$$q_{\text{str}}=\sum_{i}\frac{z_{i}e}{1+10^{z_{i}\left(\text{pH}-\text{p}K_{i}\right)}}.$$ (1)

Here *i* denotes each ionizable group, $\text{p}K_{i}$ is the negative logarithm of its acid dissociation constant, *z*_*i*_ = +1 or −1 indicates the formal valence of charge of a basic or an acidic group, respectively, and *e* is the elementary charge. In practice however, collective interactions in a densely packed system of charges can dramatically modify the molecule’s effective charge in solution via two separate phenomena, namely, charge regulation and charge renormalization. The former concerns an alteration in the charged state of an ionizable group in the context of the molecular environment, while the latter deals with the highly non-linear screening of molecular charge by counterions in the surrounding electrolyte phase. Both phenomena generally result in a reduced “effective” charge of an electrically charged object, and have each received extensive theoretical attention, from polyelectrolytes and proteins to colloidal particles and charged surfaces in solution.^7–17^ Nonetheless, experimental situations, particularly those involving proteins, would be expected to entail contributions from both charge regulation and renormalization. As a result, a theoretical analysis focusing on one or the other phenomenon will not necessarily lead to fruitful comparisons with experiment. Previous work has used mean field electrostatics to simultaneously address both the chemical reaction aspect of charge creation and charge renormalization for colloidal spheres carrying surface charge.^18^ Efforts have also been directed at calculating forces between charge regulating flat surfaces and spheres in solution.^15,19^ At the molecular level, atomistic simulation-based approaches can be used to determine the regulated charge^20,21^ and calculate interaction free energies between arbitrary macromolecules but are often resource-intensive and time-consuming.

To our knowledge, few studies thus far have integrated both charge regulation and renormalization phenomena into a comprehensive theoretical picture of an electrical charge for a generic macromolecule and its interactions, using mean field theory alone. An approach that uses mean field theory and yet permits the incorporation of a more fine-grained level of chemical and geometric detail on the macromolecule would be useful for comparison with experiments. Here we describe a finite element based numerical mean field electrostatic model of macromolecular electrostatics based on the non-linear Poisson-Boltzmann (PB) equation. In conjunction with interaction free energy calculations, we present a coherent picture of macromolecular electrostatics that includes the effects of both charge regulation and renormalization. We show that charge regulation deals with the true charge of an object in solution—a physically straightforward quantity representing the net electrical charge actually carried by ionized groups in the molecular context, depending on the pH and salt concentration in solution. The renormalized charge in turn is an effective quantity that manifests physically, for example, in the context of an interaction of the molecule of interest with another charged entity. Our approach thus not only enables quantitative predictions of the true net structural charge of macromolecules but can also be used to determine their effective or renormalized charge in solution. The model takes into account of 3D molecular geometry, finite-size effects in linear polyelectrolytes, the spatial distribution of charged groups within a molecule and can also be adapted to perform $\text{p}K_{\text{a}}$ shift calculations.

The manuscript is organized as follows: in Section II A, we focus on general principles of charge regulation in a spherical dielectric medium representing a globular macromolecule and derive a rule of thumb criterion for the onset of charge regulation. In Section II B, we lay out the governing equations in our model and validate the results against analytical expectations. Section III A examines and explains charge renormalization in the context of electrostatic interaction free energies and is followed by a comparison of results of our model to previous theoretical predictions of effective charge. Finally, Section IV compares our model predictions with a variety of experimental data. We find excellent agreement between the model predictions and experimental measurements of the effective charge of linear polyelectrolytes such as double-stranded DNA and disordered proteins, with no tunable parameters. Using the effective internal dielectric constant as a tunable parameter, we further demonstrate that the model predicts net charge values that agree well with measurements of globular macromolecules such as the tetrameric protein *β*-Glucuronidase (Gus*β*—290 kDa). Further the net charge predictions agree well with those from a fully atomistic approach both for the large multimeric Gus*β* as well as for the small monomeric lysozyme (14.3 kDa).^22^ Finally, we modify our approach to incorporate a variable local dielectric constant in order to model $\text{p}K_{\text{a}}$ shift measurements in proteins. We specifically consider recent measurements of $\text{p}K_{\text{a}}$ shifts in variants of SNase containing single amino acid substitutions.^23,24^

## II. A COMPREHENSIVE MODEL OF MACROMOLECULAR ELECTROSTATICS I: CHARGE REGULATION

### A. Charge regulation in globular macromolecules and linear polyelectrolytes—Analytical considerations

Our model regards a globular protein as a uniform dielectric sphere housing a uniform spatial distribution of ionizable groups, unless otherwise noted. These groups acquire or lose charge via protonation/de-protonation equilibria that depend on experimental conditions such as electrolyte ionic strength and pH. The charge of an isolated ionizable group in solution is a function of the group’s acid dissociation constant ($K_{\text{a}}$), solution pH, and local dielectric environment, as reflected in Eq. (1).

For a hypothetical molecule carrying identical ionizable groups on its surface, interaction among the solvent-exposed groups would lead to a local non-zero surface electrical potential, $\psi_{\text{s}}$. Since the chemical potential of the protons is constant throughout the system, the non-zero potential in the vicinity of the groups results in a local pH, different from that in the bulk. The greater the magnitude of electrical potential at the charged groups, the stronger the departure of their degree of ionization from that in isolation, as given by the following equation:^15,25^

$$\alpha =\frac{1}{1+10^{z_{i}\left(\text{pH}-\text{p}K\right)}\text{exp}\left(z_{i}e\psi_{\text{s}}/k_{\text{B}}T\right)}.$$ (2)

This behaviour essentially embodies the phenomenon termed “charge regulation.”

In our model, the sphere representing the protein is transparent to the protons in solution but impervious to the salt ions which remain in the external electrolyte phase. The protein thus acquires a net charge in solution that is the sum of the charges of its individual ionizable groups resulting from ionization equilibria modified by the local environment and intramolecular coulomb interactions. For an ionizable group embedded at a location **r** within a dielectric medium of uniform dielectric coefficient $\varepsilon_{\text{p}}$, Eq. (2) can be modified as follows:

$$\alpha \left(𝐫\right)=\frac{1}{1+10^{z_{i}\left(\text{pH}-\text{p}K\right)}\text{exp}\left\{\frac{z_{i}e\psi \left(𝐫\right)+\phi_{\text{s}}+\phi_{0}}{k_{\text{B}}T}\right\}},$$ (3)

where ψ(𝐫)represents the local electrical potential at the ionizable group and $\frac{\phi_{\text{s}}}{k_{\text{B}}T}=\frac{l_{\text{B,m}}}{2r_{{\text{A}}^{-}}}\left(\frac{\varepsilon_{\text{m}}}{\varepsilon_{\text{p}}}-1\right)\geq 0$ represents the difference in the solvation energy of the ionized group between the exterior electrolyte and interior dielectric environment. Here $l_{\text{B,m}}=\frac{e^{2}}{4\pi \varepsilon_{\text{m}}\varepsilon_{0}k_{\text{B}}T}$ is the Bjerrum length in a medium of a dielectric constant, $\varepsilon_{\text{m}}$, $r_{{\text{A}}^{-}}$ is the molecular radius of the ionized group and $\phi_{0}$ can be used to incorporate additional non-electrostatic energy contributions to the ionization equilibrium but is set to zero in this work. For example, for $\varepsilon_{\text{p}}=40$, $\varepsilon_{\text{m}}=78.5$ and taking $r_{{\text{A}}^{-}}=0.25$ nm for the ionized carboxyl group, we obtain $\frac{\phi_{\text{s}}}{k_{\text{B}}T}=1.4$.

Analytical considerations based on the volume-averaged interior potential of a dielectric sphere can be used to arrive at a threshold charge criterion that defines the onset of charge regulation for a molecule in solution. For an initial analysis of limiting behavior, we consider a hypothetical globular protein carrying only acidic groups so that *z*_*i*_ = −1. Inspection of Eq. (3) suggests an expression for a threshold local electrical potential, $\left|\psi_{\text{t}}\right|$ above which the net charge of a given ionizable group, and by implication that of the macromolecule as a whole, may begin to depart from the structural value, Q e. Denoting the total number of charges on the molecule at the threshold as $Q_{\text{t}}$ and defining $\alpha_{\text{t}}=\eta_{\text{t}}=\frac{Q_{\text{t}}}{Q}<1$, we have

$$\left|\psi_{\text{t}}\right|=\frac{k_{\text{B}}T}{e}\left|2.303\left(\text{pH}-\text{p}K_{\text{a}}\right)+\ln\left(\frac{1}{\eta_{\text{t}}}-1\right)\right|-\frac{\phi_{\text{s}}}{e}.$$ (4)

In order to estimate the magnitude of $\psi_{\text{t}}$ within a protein, we consider a sphere of radius *R*, composed of a material of dielectric coefficient $\varepsilon_{\text{p}}$, enclosing a uniformly distributed total charge Q e which in this analysis synonymous with $q_{\text{str}}$ for a molecule of known chemical composition. The charge has a uniform density, *ρ*, and the sphere is bathed in a monovalent electrolyte of concentration, *c*, in moles/liter. The electrolyte is characterized by an inverse Debye length, $\kappa =\sqrt{\frac{2N_{\text{A}}ce^{2}}{\varepsilon_{\text{m}}\varepsilon_{\text{0}}k_{\text{B}}T}}$, where $N_{\text{A}}$ is the Avogadro’s number, $\varepsilon_{\text{m}}=78.5$ is the dielectric constant of water, and $\varepsilon_{\text{0}}$ is the permittivity of free space. The local internal electrical potential of the charged sphere under consideration has contributions both from the charge distributed in its interior and from the surface potential and is given by $\psi \left(r\right)=\left(\frac{\rho }{3\varepsilon_{\text{p}}\varepsilon_{0}}\right)\left(\frac{R^{2}-r^{2}}{2}\right)+\psi_{\text{s}}$. Here $\psi_{\text{s}}$ is the electrical potential at the surface of the sphere that results from screening of the molecule’s charge by counterions in the bulk electrolyte, where the potential at an infinite distance from the molecule, $\psi_{\infty }=0$. In the linear regime, $\psi_{\text{s}}$ is well approximated by $\frac{Ql_{\text{B,w}}}{R\left(1+\kappa R\right)}$, while the volume-averaged dimensionless interior potential of the sphere, $\frac{e{\left\langle \psi \left(r\right)\right\rangle }_{\text{v}}}{k_{\text{B}}T}$, in turn works out to $\frac{Ql_{\text{B,p}}}{5R}$.

In order for charge regulation to take place, we postulate that the magnitude of the average interior potential of the sphere, $\psi_{\text{int}}=\psi_{\text{s}}+{\left\langle \psi \left(r\right)\right\rangle }_{\text{v}}$ equals or exceeds the threshold potential in Eq. (4). We thus arrive at the following threshold charge criterion for the onset of charge regulation in our representative spherical charge distribution:

$$\frac{\left|Q_{\text{t}}\right|}{R}\left\{\frac{l_{\text{B,p}}}{5}+\frac{l_{\text{B,w}}}{\left(1+\kappa R\right)}\right\}\geq \frac{e\left|\psi_{\text{t}}\right|}{k_{\text{B}}T}.$$ (5)

At pH 7 and $\text{p}K_{\text{a}}=4$, for $\eta_{\text{t}}=0.95$, $\phi_{\text{s}}=0$, and for small values of $\left|\psi_{\text{s}}\right|\approx 2$ $k_{\text{B}}T$, which is a reasonable estimate at high salt concentrations, we thus obtain

$$\frac{\left|Q_{\text{t}}\right|l_{\text{B,p}}}{R}∼10$$ (6)

This relation could serve as a rule of thumb criterion for the onset of charge regulation in a globular molecule in solution. Including an anion solvation energy contribution of $\phi_{\text{s}}=1.4k_{\text{B}}T$ would shift this threshold even lower to $\frac{\left|Q_{\text{t}}\right|l_{\text{B,p}}}{R}<5$. Still larger values of $\phi_{\text{s}}=10k_{\text{B}}T$ encountered for $\varepsilon_{\text{p}}=10$, would imply that ionization is only relevant for $\text{pH}-\text{p}K_{\text{a}}\geq 5$, and that otherwise interior ionizable groups will be completely discharged.

The result in Eq. (6) is similar to the renormalization result for spheres carrying a surface charge,^8^ except that here $l_{\text{B,p}}$ characterizes the sphere interior whereas in the renormalization approach $l_{\text{B}}$ is a property of the exterior solvent. The simple analytical result for a sphere thus suggests that charge regulation could be expected to occur even in very weakly charged globular proteins, say Q<3 and R∼1 nm. In practice however, the extent of charge regulation will depend strongly on the pH and ionic strength of the solution, $\text{p}K_{\text{a}}$ of the ionizable groups, and the 3D geometry of the molecule, the exact spatial distribution of charged groups within the molecule and additional molecular structural details that contribute to local electrical polarizability of the molecule. The ability to readily incorporate many of these descriptive features into our numerical model described below renders the framework immediately applicable to real experimental situations.

### B. Numerical approach to calculating the regulated charge of a globular macromolecule or a linear polyelectrolyte

**FIG. 1.**
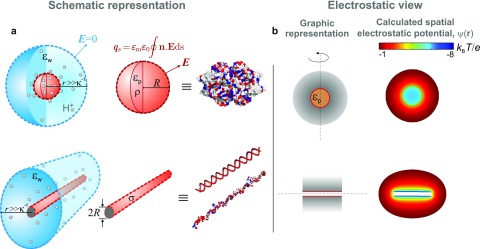
(a) Schematic illustration of our model of a charged globular macromolecule (red sphere) and a linear polyelectrolyte (red cylinder) immersed in an electrolyte (blue). Grey spheres denote protons both in free solution and in the interior dielectric environment of the globular molecule. The surface integral of an electric field, ***E***, over the sphere or cylinder yields the true net (regulated) charge of the molecule, $q_{\text{s}}$. Far away from the molecule, the corresponding surface integral in the electrolyte goes to zero, as a result of electroneutrality within the domain enclosing both molecule and electrolyte. ρ and σ—volumetric and surface charge densities in the spherical and cylindrical cases, respectively; $\varepsilon_{\text{p}}$ and $\varepsilon_{\text{m}}$—static dielectric constants of the protein interior and external electrolyte, respectively; $\kappa^{-1}$—Debye length; *R*—radius of the sphere or cylinder representing the molecule. (b) Left: Graphic representation of a spherical and cylindrical molecule in 2D. The dashed line denotes the axis of cylindrical symmetry and the grey shaded region depicts the counterion density surrounding the molecule. Right: Spatial electrostatic potentials, ψ(𝐫) for a spherical and cylindrical molecule calculated using the non-linear PB equation. The cases presented are those of Gus*β* (top) and 60 bp dsDNA (bottom) in 1 mM monovalent salt, pH 8.8.

We proceed to compare the above analytical charge regulation threshold criteria with numerical calculations of the same quantity for a spherical charge distribution representing a protein immersed in an electrolyte (Fig. 1). In order to do so, we build our model based on a “discretized continuum” approach,^20,26–28^ where at the simplest level the protein is represented by a sphere of radius *R*, whose total structural charge is given by

$$q_{\text{str}}=\sum_{i}\int_{0}^{R}\frac{z_{i}\rho_{i}}{1+10^{z_{i}\left(\text{pH}-\text{p}K_{i}\right)}}4\pi r^{2}d\text{r}.$$ (7)

Here $\rho_{i}$ represents a uniform volumetric density of charged species, *i*. Since the charge of an ionizable group depends on the local electrical potential according to Eq. (3), the local charge density in the globular distribution carrying both acidic and basic groups is given by $\rho \left(𝐫\right)=\sum_{i}z_{i}\alpha_{i}\left(\text{𝐫}\right)\rho_{i}$ where $\alpha_{i}$ denotes the fractional ionization of an acidic or basic group with a proton dissociation constant, *K*_*i*_. Note that in our model, electrolyte ions remain in the aqueous phase and are not permitted to enter the dielectric sphere representing the globular protein.

In order to obtain the spatial electrostatic potential, ψ(𝐫), in the entire system, given a set of parameter values for pH, *c*, and $\varepsilon_{\text{p}}$, we numerically solve the governing differential equations for each spatial domain (Fig. 1), subject to boundary conditions as detailed below:

Globular (sphere) interior (Poisson equation):

$$\Delta \psi \left(𝐫\right)=-\frac{\rho \left(𝐫\right)}{\varepsilon_{\text{p}}\varepsilon_{0}}.$$

Electrolyte phase (Poisson-Boltzmann equation):

$$\Delta \psi \left(𝐫\right)=\kappa^{2}\sinh\psi \left(𝐫\right)$$

Boundary conditions for the electric field, **E**, are as follows:

1. Overall electroneutrality: **n**.**E** = 0 for $r∼R+10\kappa^{-1}$
2. Regulating surface charge (e.g., for cylindrical polyelectrolytes):

$$𝐧.\varepsilon_{\text{m}}\varepsilon_{\text{0}}𝐄\left(𝐑\right)=\sigma \left(𝐑\right)=\sum_{i}z_{i}\alpha_{i}\left(𝐑\right)\sigma_{i},$$

where $\sigma_{i}\left(𝐑\right)$ is the local charge density due to ionizable species *i*, **r** = **R** denotes the cylinder’s surface, and **n** is the outward pointing surface normal. Note that ρ(𝐫) and σ(𝐑) above are themselves functions of ψ(𝐫) as given by Eq. (3).

We describe the electrolyte phase with a uniform dielectric coefficient of $\varepsilon_{\text{m}}=78.5$ representing water at 25 °C, and pH and bulk salt concentration, *c*, corresponding to the experimental conditions. Similar to traditional treatments, our dielectric sphere is impervious to salt ions in the aqueous phase.^20,26–28^ But by contrast, protons are permitted to penetrate the sphere, enabling us to account for the pH- and interior dielectric coefficient-dependent titration of charged groups in a single calculation. The transparence to protons of our sphere representing a globular protein seems justified on account of independent experimental observations such as the well known pH-dependent emission of the buried chromophore in green fluorescent protein,^29^ measured pH-dependent NMR shifts of ionizable interior groups, and the alteration of protein stability due to titration of internally buried charged groups,^23,24,30^ to name a few. The principle is also commonly used in atomistic modelling of the protonation of interior ionizable groups in proteins.^21,22,31^

Furthermore we treat linear polyelectrolytes, polypeptide chains, and single-stranded and double-stranded nucleic acids as rigid, hollow cylinders of diameters, *D* = 0.5 nm, 1 nm, and 2 nm, respectively, whose lengths, *L*, correspond to full extension. We consider the charge of the molecule as a uniform density, $\sigma_{i}$, distributed all over the cylindrical surface representing the molecule, unless otherwise required. Importantly, since the ionizable groups here are fully exposed to the exterior electrolyte phase, ψ(𝐫) in Eq. (3) is just the local surface potential on the cylinder and the “interior dielectric coefficient,” $\varepsilon_{\text{p}}$ is not a relevant model parameter.

We numerically solve the governing equations subject to the relevant boundary conditions using COMSOL Multiphysics. In almost all cases, except that of inhomogeneously charged molecules, a 2D axisymmetric model suffices to describe the molecule. The geometry was meshed using a triangular mesh which was refined until the computed free energy values converged. A typical value for the maximum linear size of a mesh element was 0.5 nm. Boundary layer meshes with at least an order of magnitude smaller element size were used at all charged surfaces where strong spatial variations in electrical potential were expected. The estimated numerical error on the computed values is less than 5%, and typical computation times were of the order of 1 s or less on a standard PC (Intel Core i3-2120 processor).

Knowing ψ(𝐫) in the whole system, we obtain the regulated charge of the molecule, $q_{\text{s}}$ by integration. Specifically, we have $q_{\text{s}}=\int_{0}^{R}\rho \left(𝐫\right)4\pi r^{2}d\text{r}$ for globular molecules and $q_{\text{s}}=\int_{0}^{L}\sigma \left(𝐑\right)2\pi \text{𝑅}d\text{l}$ for cylindrical polyelectrolytes. We emphasize that $q_{\text{s}}$ includes the effect of the local electrical potential on the ionization equilibrium of each group and is therefore different from the nominal structural charge, $q_{\text{str}}$ defined in Eq. (7).

Our numerical finite-element based computational approach goes much beyond predicting charge regulation threshold criteria; it also enables a direct, rapid (<1 s) estimate of the true net charge of the molecule given experimental conditions such as pH, solution ionic strength, molecular geometry, amino acid composition, and spatial charge distribution patterns, in one direct computational step. The approach does not require the implementation of free energy cycles, which can turn cumbersome for molecules with many charged groups.^20^ When the calculation is fine-tuned against a measured quantity such as the effective charge or isoelectric point of a globular molecule in solution, the model also yields an estimate of the interior dielectric coefficient (as described later). The dielectric coefficient in turn reflects the density of atomic packing in the molecule or in other words, the folded state of a macromolecule such as a protein.

**FIG. 2.**
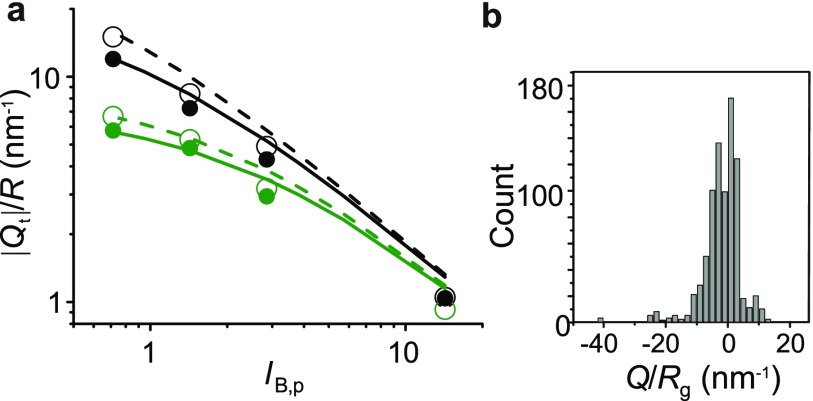
(a) Comparison of the numerically calculated and analytically determined threshold charge, $Q_{\text{t}}$ corresponding to $\eta_{\text{t}}=0.95$ at which the onset of charge regulation occurs in a spherical charge distribution of radius, *R*, with $\phi_{\text{s}}=0$. Symbols denote calculations and lines denote the result of Eq. (5). Color denotes different salt concentrations: ∼100 mM (black) and ∼1 mM (green). Open symbols and dashed lines denote *R* = 5.1 nm, while closed symbols and solid lines represent *R* = 2.55 nm. (b) Histogram of $Q/R_{\text{g}}$ values for ∼11 000 globular monomeric proteins, where $R_{\text{g}}$ denotes the calculated radius of gyration of each molecule based on its atomic structure.

**FIG. 3.**
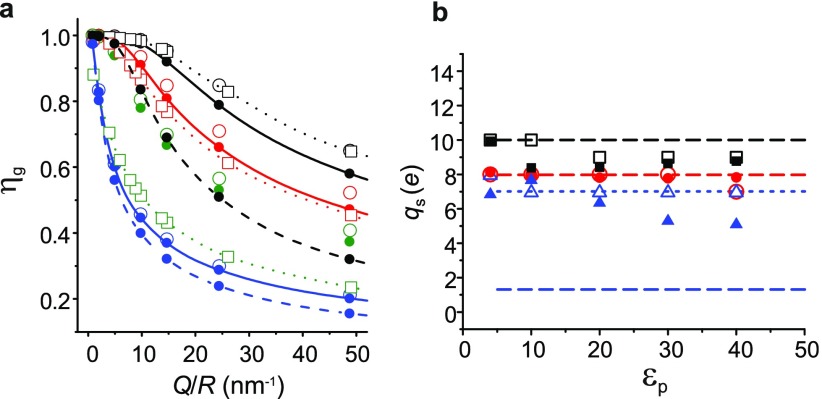
(a) Calculated charge regulation factor, $\eta_{\text{g}}$ as a function of *Q*/*R* for spherical charge distributions of radii, *R* = 5.1 nm (open symbols) and 2.55 nm (closed symbols) and various values of the interior dielectric constant, $\varepsilon_{\text{p}}=78.5$ (black), 40 (red), 20 (green), and 4 (blue). Square symbols represent calculations including the contribution of the Born solvation energy term, $\phi_{\text{s}}$ (see Eq. (3)), but $\phi_{\text{s}}=0$ for all other cases. All lines are guides to the eye. c∼100 mM, pH = 7 and $\text{p}K_{\text{a}}=4$ for all calculations except the dashed lines presented for $\varepsilon_{\text{p}}=78.5$ and 4 for which c∼1 mM. (b) Comparison of the net charge, $q_{\text{s}}$ of the protein lysozyme predicted by our model (closed symbols) and an atomistic calculation using the H++ platform (http://biophysics.cs.vt.edu/H++, version 3.2) (Ref. 22) (open symbols), at pH = 5 (black), 7 (red), and 9 (blue), as a function of $\varepsilon_{\text{p}}$. Dashed and dotted lines represent nominal values from Eq. (1), including and excluding the contribution of the 8 cysteine residues in lysozyme, respectively.

The results of the numerical model confirm analytical considerations and show that charge regulation in globular proteins is expected under a wide range of conditions (Fig. 2(a)). Fig. 3(a) presents the calculated fractional charge regulation, $\eta_{\text{g}}=q_{\text{s}}/eQ$ at pH 7 for a dielectric sphere housing *Q* acidic groups of $\text{p}K_{\text{a}}=4$, as a function of *Q*/*R*. Comparing the calculated $\eta_{\text{g}}$ values over a wide range of *Q*/*R* for different values of $\varepsilon_{\text{p}}$ at low (1 mM) and high (100 mM) salt concentrations reveals an interesting feature, namely, the lower the interior dielectric constant, the weaker the influence of the salt concentration in the electrolyte on $\eta_{\text{g}}$. The reason is evident upon recognizing that for low values of the interior dielectric coefficient, the dominant contribution to the interior electrical potential arises from strong electrostatic coupling of the charges within the low dielectric medium. Here, the surface potential of the sphere, $\psi_{s}$, which reflects the electrolyte environment, is generally small in comparison to the interior potential and thus does not have much of an effect on $\eta_{\text{g}}$. On the contrary, for high values of $\varepsilon_{\text{p}}$, say ≥40, decreasing *c* has a much greater effect on $\eta_{\text{g}}$ and can lower it by up to a factor of 2, as the contribution of $\psi_{\text{s}}$ to $\psi_{\text{int}}$ is now higher in a relative sense. Thus the model predicts that a compactly folded protein with *Q*/*R* = 2.5 ${\text{nm}}^{-1}$ and charged groups distributed in a medium of the interior dielectric constant $\varepsilon_{\text{p}}=20$ experiences 78% charge regulation in a solution containing 100 mM monovalent salt at pH 7 (open green square symbols in Fig. 3(a)). This could, e.g., refer to a molecule of radius *R* = 1 nm carrying a net structural charge of 2.5 *e*. An analysis of $Q/R_{\text{g}}$ values of ∼11 000 globular monomeric proteins and ∼800 globular multimeric proteins in the Protein Data Bank (PDB) reveals that >50% and >65%, respectively, fall in the range *Q*/*R* = 2.5 ${\text{nm}}^{-1}$ (Fig. 2(b)). This strongly suggests that charge regulation could be a ubiquitous effect, important to consider in accounting for the electrostatic interactions of all but the most weakly charged globular macromolecules.

Finally, Fig. 3(b) presents a comparison of the net charge, $q_{\text{s}}$, of the protein lysozyme (PDB code: 1W08) predicted by this model and an atomistic calculation using the H++ platform^22^ (http://biophysics.cs.vt.edu/H++, version 3.2) which employs the methodology of Bashford and Karplus.^20^ In our model, we exclude the charge contribution of the 8 cysteine side-chains ($\text{p}K_{\text{a}}≃8.2$) as lysozyme is known to possess 4 native disulfide bonds. The values of molecular charge predicted by the two approaches agree within 20% over a wide range of pH and $\varepsilon_{\text{p}}$. Thus, despite the lack of atomistic detail, our model is able to capture essential features of the underlying electrostatics. The results further suggest that our model could additionally permit facile interrogation of the net charge of a molecule in response to deletion or inclusion of specific charged residues, which may not be straightforward using the atomistic approach.

Once the net (regulated) charge of a molecule is known from the above calculation, we may question the interaction free energy of the molecule with another entity, say a like-charged plate. In fact the magnitude of this interaction energy leads us to a new definition of the effective or renormalized charge of the molecule as described below.

## III. A COMPREHENSIVE MODEL OF MACROMOLECULAR ELECTROSTATICS II: CHARGE RENORMALIZATION

### A. Using interaction free energies to calculate charge renormalization

Charge renormalization, or counterion condensation, has been known for at least 50 years in linear polyelectrolytes.^7^ More recently it has been theoretically shown to be relevant not only for charged cylinders but also for other geometries such as spheres and planes.^10,32^ The phenomenon at the heart of charge renormalization is the highly non-linear spatial distribution of the counterions enveloping a strongly charged entity in solution. Electroneutrality requires that the charge carried by the object is exactly balanced by counterions both loosely and tightly held in the surrounding electrolyte phase. For a highly charged object, the counterion-object interaction energy can be much larger than $k_{\text{B}}T$ very close to the surface. Thus the fraction of counterions closest to the object and interacting strongly with it are in some sense “unavailable” to participate in physical processes, where they would otherwise result in measurable differences in experimentally accessible quantities. From a “far-field” perspective, the strongly interacting counterions may also be thought to neutralize a portion of the charge carried by the object. As a result, various physical properties sensitive to the activity of the counterions are predicted, and even observed in experiment, to behave as if the object carried an “effective charge” much smaller than its structural charge.^11^ For example, in an electrostatic interaction, a highly charged entity would behave like it carried a much lower, renormalized or effective charge, $q_{\text{eff}}$. In fact, the far-field (κr≫1) electrostatic interaction energy between two charged spheres in the regime κR≪1 is given by the screened coulombic form,

$${\left(\frac{q_{\text{eff}}}{e}\right)}^{2}l_{\text{B}}\frac{\exp\left(-\kappa r\right)}{r},$$ (8)

where the prefactor is an effective charge, $q_{\text{eff}}$, rather than the true charge *Q e* of the spheres.^11^

There are many different approaches to calculate $q_{\text{eff}}$, the effective charge of an object in solution. These range from a far-field electrical potential matching procedure,^8,9,33^ to more thermodynamic definitions involving free energy minimization in the partitioning of ions between the condensed and free states,^10,30^ as well as estimates based on equivalent osmotic pressures in particle mixtures and diffusion coefficients of counterions.^11^ In general all of these approaches yield comparable but not identical results, which has made it difficult to provide a unique physical definition of the renormalized charge.

The quantity that is not only readily measurable in our experiments,^34^ but of general importance in the interaction of a molecule with its environment, is an interaction free energy. We therefore introduce the electrostatic interaction free energy as a means to determining an effective or renormalized charge that can not only be directly measured in experiment but also readily calculated and therefore compared with predictions of effective charge calculated using other approaches.

We solve the non-linear Poisson-Boltzmann equation and calculate interaction free energies, $F\left(z^{*}\right)$ at a fixed inter-surface separation, $z^{*}\geq \kappa^{-1}$ between a charged object of interest situated midway between parallel flat plates carrying uniform, constant surface charge, as previously described.^35^ In particular we use the following expression for the electrostatic free energy of a charge distribution, derived previously by Overbeek^36^ and implemented recently for our parallel-plate system:^35^

$$F\left(z^{*}\right)={\int_{V}\left\{\frac{\varepsilon_{\text{m}}\varepsilon_{0}}{2}\left(\text{𝑬}\cdot \text{𝑬}\right)-2c_{0}k_{\text{B}}T\left(-\psi \sinh\psi +\cosh\psi -1\right)\right\}dV|}_{z^{*}}.$$ (9)

**FIG. 4.**
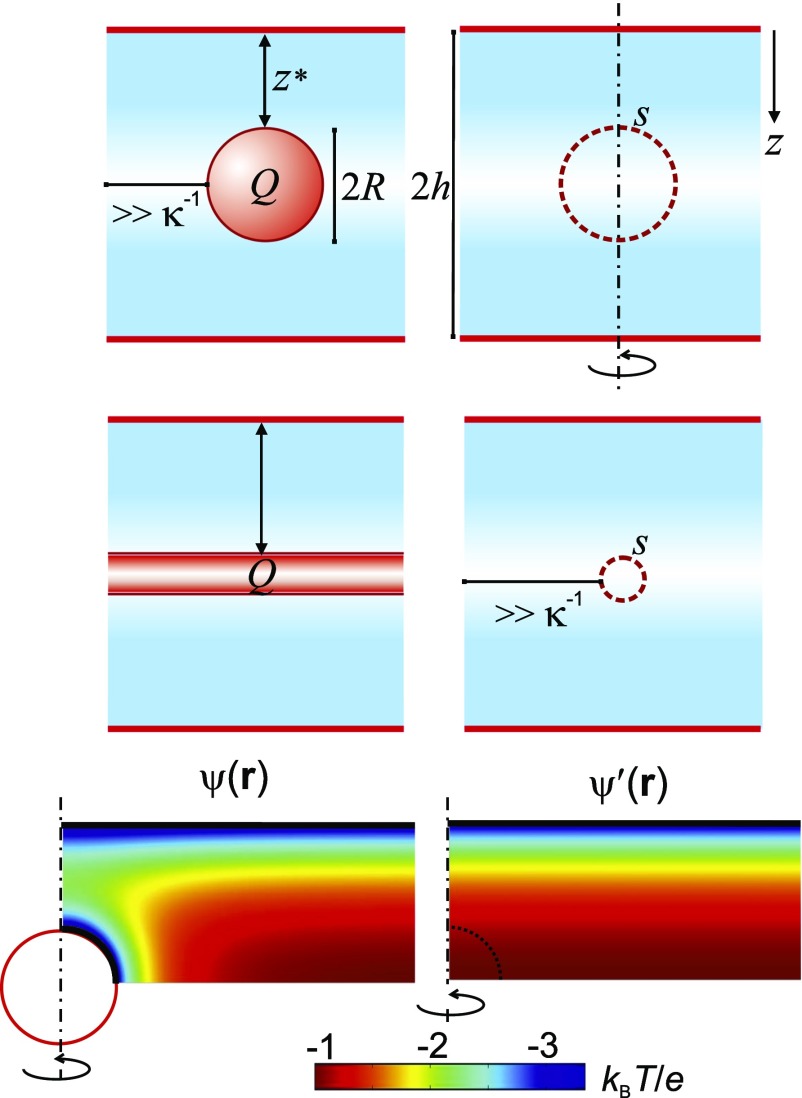
Schematic depiction of the interaction free energy method to calculate charge renormalization in macromolecules. The electrostatic potential distribution in an electrolyte-filled parallel-plate slit of height 2*h* is obtained by solving the non-linear Poisson-Boltzmann equation using constant charge boundary conditions, both with (left) and without the object (right). The average electrical potential ${\left\langle \psi^{\prime }\left(s\right)\right\rangle }_{z^{*}}$ in the slit is evaluated over the dashed circular contour representing the “virtual” surface of an object placed at $z=z^{*}+R=h$ (right). Electrostatic interaction free energies, $F\left(z^{*}\right)$, are calculated for an object of charge, *Q*, and radius, *R*, located at the mid-plane of the slit, at an inter-surface separation, $z^{*}=h-R$, using the ψ(𝐫) distribution (left) and Eq. (9). Dashed vertical lines denote axes of cylindrical symmetry.

The slit surfaces are composed of like-charged walls and create a potential distribution in the gap, which in the absence of the molecule and far away from the walls is well approximated by the expression, $\psi \left(z\right)=\psi_{\text{wall}}[\exp\left(-\kappa z\right)+\exp\left(-\kappa \left(2h-z\right)\right]$(Fig. 4). We determine two quantities: (1) the average of the electrical potential, $\psi^{\prime }\left(s\right)$ due to the slit alone over a virtual contour *s* representing the object’s surface (dashed curves in Fig. 4), for values of inter-surface separation $z^{*}∼\kappa^{-1}$, and (2) the object-slit interaction free energy, $F_{\text{os}}\left(z^{*}\right)=F\left(z^{*}∼\kappa^{-1}\right)-F\left(z^{*}∼\infty \right)$ using Eq. (9).

Comparing the calculated object-slit interaction free energy, $F_{\text{os}}\left(z^{*}\right)$, with the electrostatic energy of an equivalent charged test object placed at $z=z^{*}+R$, we find that the former is almost always significantly smaller than the latter, in keeping with the expectation created by the charge renormalization concept. Note that our test entity has the same geometry, spatial charge distribution, and total charge, *Q*, as the object of interest but does not perturb the spatial electrostatic landscape in which it is placed. In other words, we find in general $F\left(z^{*}\right)<eQ{\left\langle \psi^{\prime }\left(s\right)\right\rangle }_{z^{*}}$.

We therefore write $F_{\text{os}}\left(z^{*}\right)$ in terms of an effective charge, $q_{\text{eff}}$, rather than the true charge, *Q*, and a local electrical potential, $\psi^{\prime }\left(s\right)$, such that

$$q_{\text{eff}}{\left\langle \psi^{\prime }\left(s\right)\right\rangle }_{z^{*}}=F_{\text{os}}\left(z^{*}\right).$$ (10)

**FIG. 5.**
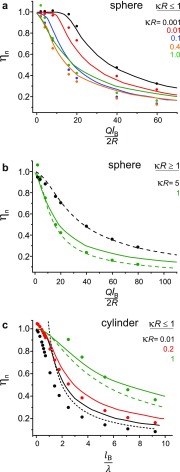
Comparison of our calculated charge renormalization factor, $\eta_{\text{n}}$, with that calculated using other approaches. Symbols denote our results; solid, dashed, and short-dashed lines represent results of Refs. 10, 9, and 7, respectively. (a) $\eta_{\text{n}}$ for spheres of radius, *R* in the regime κR≤1. (b) spheres in the regime κR≥1. (c) Cylinders in the regime κR≤1.

Thus $q_{\text{eff}}$ may be thought to represent an “interaction charge.” Interestingly we find that $q_{\text{eff}}$ agrees well with the values of an effective or renormalized charge calculated using charge renormalization and counterion condensation theories^7,9,10,35^ (Fig. 5). Importantly, when *Q* is small, as for a weakly charged entity, we find that $F\left(z^{*}\right)=eQ{\left\langle \psi^{\prime }\left(s\right)\right\rangle }_{z^{*}}$, implying no charge renormalization.

Furthermore we have verified that in the regime κR≤1, Eq. (10) can be used to describe the interaction energy—and therefore also the repulsive force—between a sphere and a single flat plate, which is a sphere of an infinite radius. Thus it is clear that our effective charge is physically the same quantity as that defined based on the Debye-Hückel interaction between two isolated spheres, where the interaction energy in the far-field is given by the product of the effective charge of one entity and the potential created by other, as shown in Eq. (8).^11^ This renormalized charge is also fundamentally the same as that obtained by the far-field potential matching procedure.^9,33^ It is also worth noting that this definition of the effective electrical charge of a molecule in solution is thus founded in the basic definition of the electrical field, ***f*** = *q**E***, where ***f*** is the force on a test charge, *q*, due to the local field, ***E***.

Finally, we point out that the object-plate separation, $z^{*}$ does not influence the calculation over a wide range, namely, $z^{*}∼1\text{–}8\kappa^{-1}$, but in general, values of $z^{*}$ satisfying $\kappa z^{*}∼3\text{–}5$ are optimal for the calculation. In other words, the calculation is not sensitive to the particular value of the local potential $\psi^{\prime }\left(s\right)$, as long as it is non-zero. In particular, in the point-object regime, where κR<1, $\psi^{\prime }\left(s\right)$ tends to the potential at a point in the slit given by the center of the object. This is an important result since it enables the spatial interaction free energy of a particle in the slit to be expressed in terms of the product of an effective charge parameter, $q_{\text{eff}}$ and the electrical potential at a point in the slit. The relation in Eq. (10) is expected to hold in general, except at separations $\kappa z^{*}\ll 1$ where the “near-field” or highly non-linear region of the counterion distribution is probed.

We further define a quantity $\eta_{\text{n}}=q_{\text{eff}}/eQ$ that denotes the extent of charge renormalization and can be directly compared with values obtained using other theoretical approaches. While it is not always clear how some of the previous definitions and calculations of $q_{\text{eff}}$ can be directly and quantitatively related to particular experimental situations, our interaction-energy-based definition of $q_{\text{eff}}$ can be readily related to measurements.^34^ Importantly, our approach supports direct incorporation of geometric and chemical information on the molecule, e.g., shape, non-uniform charge distribution, finite length in polyelectrolytes, which is vital for comparison with experiments.

### B. Charge renormalization in spheres and cylinders

We calculate electrostatic free energies of interaction, F(x) and determine the effective charge, $q_{\text{eff}}$ of charged spheres and cylinders by the procedure described above, and illustrated in Fig. 4. For this analysis, we bestow on all surfaces, namely, those of the sphere or cylinder of interest and the parallel flat plates—a uniform, constant surface charge. Interaction free energies are calculated for various values of object charge, *Q*, geometric parameters, *R* and *x*, as well as salt concentrations, *c* as previously described.^35^

In order to directly relate our results to other theoretical approaches, we consider spheres and cylinders of fixed surface charge, i.e., the charge regulation aspect of the model, described in Sections II A and II B, is switched off. Fig. 5 compares our calculated values of $\eta_{\text{n}}$ for objects of constant charge with the values obtained in three other theoretical approaches, namely, those of Manning, Netz, and Aubuoy *et al.* Manning’s prediction for one-dimensional polyelectrolytes is supposed to be valid for all salt concentrations (dashed line in Fig. 5(c)). Aubouy *et al.* provides calculations for spheres and cylinders of radius *R* in the regime κR≥1.

Overall there is good to excellent agreement between our calculations and previous work. Interestingly we find that for spheres, good agreement can be expected over a wide range of κR=0.001 to 5 (Figs. 5(a) and 5(b)). For example, κR∼5 corresponds to a relatively large globular macromolecule, 5 nm in radius in 90 mM monovalent salt.

We further calculate $\eta_{\text{n}}$ as a function of $l_{\text{B}}/\lambda$, for infinite cylinders of radius, *R* and linear charge density, λ. We find reasonably good agreement in the regime κR≤1, with the regime κR>1 generally resulting in $q_{\text{eff}}>q_{\text{str}}$ for the lower range of $l_{\text{B}}/\lambda$ values. In Fig. 5(c), for example, for κR=1 and $l_{\text{B}}/\lambda <2$, we find that the calculated interaction free energy exceeds the expectation based purely on the electrostatic argument in Eq. (9) with $q_{\text{eff}}=q_{\text{str}}$, yielding $\eta_{\text{n}}>1$. In contrast, for strongly charged polyelectrolytes in the regime $l_{\text{B}}/\lambda >2$ (for example, dsDNA has $l_{\text{B}}/\lambda =4.2$), the rms deviation between our calculation and the nearest other theoretical prediction is <10%.^7,9,10^ Note that the κR<1 regime should indeed well describe one-dimensional macromolecules under most experimental conditions of interest. For example, in 100 mM monovalent salt, where $\kappa^{-1}=0.9$ nm, *R* ranges from 0.25 nm for polypeptide chains to 1 nm for dsDNA. Thus for most polyelectrolytes, κR substantially exceeds 1 only for c≫100 mM, where the Debye length approaches the Bjerrum length, ion-ion correlations become important and it is not clear that PB theory retains general validity in this limit.^37^

To conclude this section, we point out that for spheres of constant charge in very low salt, counterion-condensation analysis predicts the re-entrant behavior for *η*.^10,32^ Here the object’s charge is renormalized at intermediate salt concentrations but increases towards its full structural value in both limits as κ→0 as well as ∞. However when both regulation and renormalization are at play, as in our full model and in actual experiments, we do not expect this non-monotonic behavior as κ→0. Decreasing *c* increases the magnitude of the sphere surface potential monotonically, which in turn exponentially damps the net charge of a regulating ionizable group driving down the sphere’s net charge, as evident from Eq. (3). Departures from this expectation are of course possible for objects composed of a mixture of ionizable groups with vastly different $\text{p}K_{\text{a}}$s.

## IV. COMPARING MODEL PREDICTIONS WITH EXPERIMENTS

### A. Effective charge measurements on biomolecules

Table I compares predictions of effective charge from our full model, including both charge regulation and renormalization, with experimental measurements of effective charge using our recently developed method, “escape-time electrometry” (ET*e*),^34^ for several classes of biomolecules such as DNA and intrinsically disordered and globular proteins.

**TABLE I.** The effective electrical charge of nucleic acids and proteins measured using ET*e* ($q_{\text{m}}$) and compared with our calculations ($q_{\text{eff}}$) and other theoretical predictions ($q_{\text{theory}}$).^34^ $q_{\text{s}}$ denotes the net (regulated) charge of the molecule. $q_{\text{str}}$ is a sequence-based estimate from Eq. (1) at the experimental pH, including the contribution of fluorescent dye moieties coupled to the molecule. For DNA, the contribution of dyes is taken as an additive in all theoretical estimates, while for proteins, $q_{\text{str}}$ remains effectively unaltered. $q_{\text{theory}}$ lists the predicted effective charge from charge renormalization theories and from an atomistic structure-based calculation for the globular protein. $q_{\text{eff}}$ values carry an estimated 1%-5% uncertainty due to numerical error. All values of the charge in units of −*e*. Reprinted with permission from Ruggeri *et al.*, Nat. Nanotechnol. **12**, 488–495 (2017). Copyright 2017 Springer Nature.^34^

				Model predictions		Measurement
	Molecule	$q_{\text{str}}$	$q_{\text{theory}}$	$q_{\text{s}}$	$q_{\text{eff}}$	q_m_
DNA	40 bp	82	30.9^a^; 21^b^	80.3	32.4	37.1 ± 0.8
	60 bp	124	48^a^; 31^b^	121.4	45.7	42.9 ± 2.5
Protein	ProT*α*	46	35^a^; 34.8^b^^,^^c^	44.5	31	28.5 ± 1.2
	Stm-l	102.7	96^a^; 102.7^b^	101.8	89.6	88.8 ± 3.5
	Gus*β*	133.8	24.3 ^d^	21.5	21.5	21.5 ± 0.9
			$\left(\varepsilon_{\text{p}}=11\right)$	$\left(\varepsilon_{\text{p}}=11\right)$		

^a^ Effective charge predictions of Ref. 10.

^b^ Effective charge predictions of Ref. 7.

^c^ Effective charge predictions of Ref. 38.

^d^ Protein net charge calculated from pK-1/2 values based on PDB structure (http://biophysics.cs.vt.edu/H++, version 3.2) (Ref. 22).

In order to determine the regulated charge, $q_{\text{s}}$, and the effective charge, $q_{\text{eff}}$, for a given biomolecule under a specific set of solution pH and salt concentration conditions, we solve the equations outlined in Section II B subject to the relevant boundary conditions. Using the obtained ψ(𝐫) distribution, we determine the regulated charge, $q_{\text{s}}$ as described. Take $Q=q_{\text{s}}$, we then determine the effective charge, $q_{\text{eff}}$ as described in Section III A. Note that in our experiments where κh> 3 in general, we find that the regulated charge, *q*_*s*_, for the molecule in the slit ($\kappa z^{*}∼3$) is essentially identical to the free solution value ($\kappa z^{*}∼10$). We therefore perform charge renormalization calculations using an expression for the free energy, *F*, corresponding to the case for a particle at constant charge, shown in Eq. (9).^35^

We find very good agreement between the measurements and calculations. Note that in our model of linear polyelectrolytes describing nucleic acids and disordered proteins, no parameters were tuned in order to obtain agreement with experiment. For globular macromolecules, however, we do tune the value of $\varepsilon_{p}$ in order to obtain an agreement with the charge measurement. We further note for a given set of experimental parameters, namely, pH and salt concentration, the calculated regulated charge, $q_{\text{s}}$, and corresponding effective internal dielectric constant predictions from our model agree well with the results of an atomistic approach for lysozyme (1W08) and Gus*β*.

### B. Estimating the interior dielectric coefficient of a globular protein

Computing the net charge of a protein in solution within the traditional atomistic approach involves correctly assigning the titration state of each group under a given set of solution conditions. The most popular methods are based on electrostatic continuum models that numerically solve the linearized PB equation. The protein is treated at the atomistic level described by a molecular mechanics force field, embedded in a uniform dielectric continuum with dielectric constants of 80 for the solvent and 4–20 for the protein interior. A $\text{p}K_{\text{a}}$ shift is calculated from the difference in electrostatic energy of a residue in its charged and neutral form and this shift is added to a model $\text{p}K_{\text{a}}$ value. Once the $\text{p}K_{\text{a}}$ shifts of all the ionizable groups have been assigned, the net charge at any given pH follows directly. For a protein housing *N* charged groups, a total of 2^*N*^ titration states need to be accounted for, requiring solution of the multiple titration site problem.^39^ For example, for a large protein such as Gus*β*, a fully atomistic calculation of net charge for a given value of the interior dielectric constant and under a specific set of experimental conditions (pH and *c*) takes several hours.

**FIG. 6.**
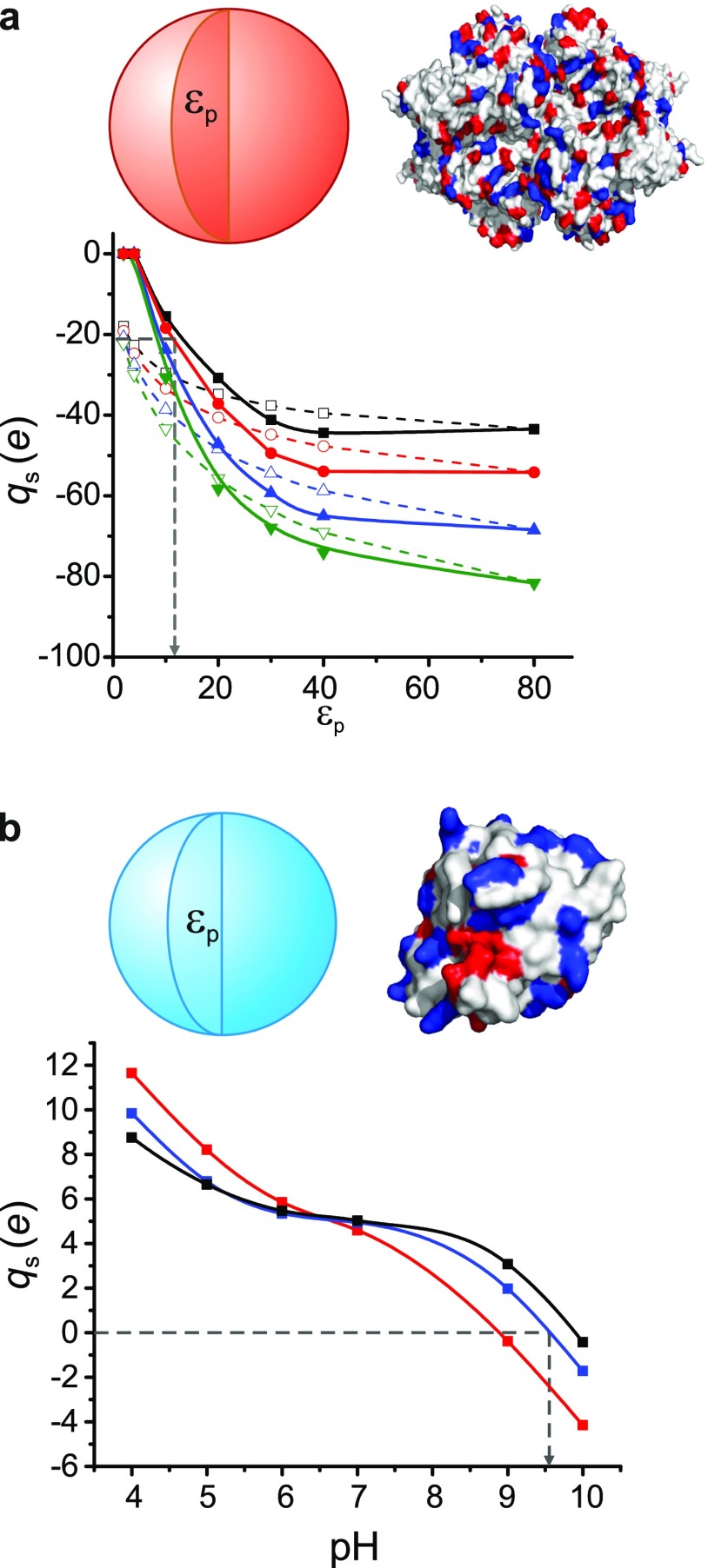
(a) Calculated net charge, $q_{\text{s}}$ for Gus*β* as a function of the interior dielectric constant, $\varepsilon_{\text{p}}$ for various salt concentrations, *c* = 0.1 mM, 1 mM, 10 mM, and 100 mM from top to bottom, and pH 8.8. Open symbols denote calculations with $\phi_{\text{s}}=0$ for comparison. The experimentally inferred value of $q_{\text{s}}=-21.5$ *e* in 1 mM monovalent salt and corresponding inferred value of dielectric constant, $\varepsilon_{\text{p}}=11$ are displayed. (b) Calculated charge of Staphyllococcus nuclease (SNase) as a function of pH in a solution of monovalent concentration, *c* = 100 mM for various values of $\varepsilon_{\text{p}}=$78.5 (black), 40 (blue), and 25 (red). The experimentally measured isoelectric point, pI = 9.6 is captured for $\varepsilon_{\text{p}}=$ 40. All lines are guides to the eye and the atomistic structures are presented with positive surface residues colored blue and negative residues, red.

Fig. 6(a) shows our theoretically predicted net structural charge, $q_{\text{s}}$ for the globular tetrameric protein Gus*β*, as a function of average interior dielectric coefficient $\varepsilon_{p}$ for cases where the Born term, $\phi_{\text{s}}$ is included and excluded. We find the experimentally measured charge of $q_{\text{m}}=-21.5$ *e* corresponds to a value of $\varepsilon_{p}=11$ for the interior of Gus*β*. Rather than a true dielectric constant, $\varepsilon_{p}$ in our simplified model is an effective parameter that is expected to capture all physical contributions to the energetics of charging that are not explicitly reflected in the model, as discussed further in Sec. IV C. The value of $\varepsilon_{p}<78.5$ in our model would therefore be expected to serve as no more than a physical indication of the molecule’s compact folded state. Nonetheless our measured charge $q_{\text{m}}=-21.5$ *e* and corresponding predicted value of $\varepsilon_{p}=11$ for the interior of Gus*β* agree well with the prediction of a fully atomistic calculation using the H++ platform (*q* = −24.3 *e* and $\varepsilon_{p}=11$) for the relevant experimental conditions. Furthermore, our calculation takes <1s for a given combination of $\varepsilon_{p}$, pH, and *c*.

### C. $\text{p}K_{\text{a}}$ shift measurements

Finally we apply our approach to model recent $\text{p}K_{\text{a}}$ shift measurements on SNase.^23,24^ In these experiments, residues at interior hydrophobic positions in the protein were substituted by glutamate residues of nominal $\text{p}K_{\text{a}}$ 4.3. The $\text{p}K_{\text{a}}$ shift of these buried ionizable groups was then experimentally obtained by measuring the pH dependence of the protein’s thermodynamic stability. $\text{p}K_{\text{a}}$ shift measurements probe the local spatially variable dielectric constant in the vicinity of the substituted residue in the protein.

Our model as described so far treats the protein as a sphere of a uniform average dielectric constant, glossing over local variations in $\varepsilon_{p}$. In order to capture local dielectric effects in SNase, we treat the protein as a sphere of radius 2.2 nm (based on the PDB structure for 3BDC) and create a separate geometric domain to account for a locally different dielectric constant around the charged group of interest. We thus treat the protein as a “core-shell” particle where the core region, which houses the single introduced glutamate group, is a sphere of radius $r_{\text{c}}=0.6$ nm, with a dielectric coefficient $\varepsilon_{c}$. This value has been chosen to represent a region in the protein interior that houses about 2 amino acids.^31^ The shell on the other hand represents the rest of the protein and is treated as a uniform dielectric as before: it has an outer radius of 2.2 nm and a uniform dielectric coefficient $\varepsilon_{s}$, which is physically the same quantity as $\varepsilon_{p}$. The shell region also carries the entire formal charge of the SNase molecule. Formally SNase is a positively charged protein and remains so up to pH>9. We solve the model for various values of pH, $\varepsilon_{s}$ and $\varepsilon_{c}$ in an electrolyte containing 100 mM monovalent salt. The calculation takes approximately 90 s.

Integrating the charge within the core region and plotting this value as a function of pH yields a theoretically predicted value of the $\text{p}K_{\text{a}}$ of the ionizable group in question. Note that the core region does not have to be centered in the sphere representing the protein but has been so chosen in the present case after verifying that off-center locations do not substantially change the results of the calculation. We also find that the calculated $\text{p}K_{\text{a}}$ shows some dependence on the radius of the core region. Decreasing the radius *r*_*c*_ of the low-dielectric core region from 0.6 nm to 0.5 nm, for example, shifts the inferred $\text{p}K_{\text{a}}$s up by ∼0.1 units. For $r_{\text{c}}=0.5\text{–}0.6$nm, varying $\varepsilon_{c}$ and $\varepsilon_{s}$ we find that the model predicts $K_{\text{a}}$ shifts in the range 4.5 to 9.1 for values of $\varepsilon_{s}=40$, and $\varepsilon_{\text{c}}$ in the range 8 to 20, in good agreement with the reported values.^23^ These values for $\varepsilon_{\text{c}}$ and $\varepsilon_{s}$ are reasonable when compared with predictions of the local dielectric coefficients from theoretical models of the interior of globular proteins.^40^

Incidentally, although on the comparatively high side, a value of $\varepsilon_{p}=40$ in our model for SNase reproduces its experimentally measured isoelectric point, pI = 9.6 (Fig. 6(b)).^41^ Importantly we further find that first principles calculations of the dielectric constant of SNase also yield a rather high value of 20-30 which has been attributed to intrinsic backbone fluctuations originating from the molecule’s structural architecture.^42^ Furthermore since in our model, $\varepsilon_{p}$ is a parameter representing the average dielectric environment over all ionizable groups in a protein, it may be reasonable to expect that smaller proteins, e.g., SNase (radius, 2.2 nm) with a larger surface to volume ratio, and therefore on average greater exposure per residue to the external electrolyte ($\varepsilon_{w}$ = 78.5), require a larger model value of $\varepsilon_{p}$ than larger proteins such as Gus*β* (radius, 5.1 nm, $\varepsilon_{p}∼11$), all other effects remaining equal. Thus, calibrating the model against, e.g., an isoelectric point measurement on a protein of interest would yield the required value of $\varepsilon_{p}$ to be input to the calculation. This parameter value would implicitly account for all effects not explicitly considered in the model. Moreover, given the atomic structure of a globular molecule, a facile initial step towards fine-tuning the spatial charge distribution in the model would be to split the charged residues into surface and interior groups and explicitly account for the solvent-exposed surface groups as illustrated in Fig. 1 for polyelectrolytes.

**FIG. 7.**
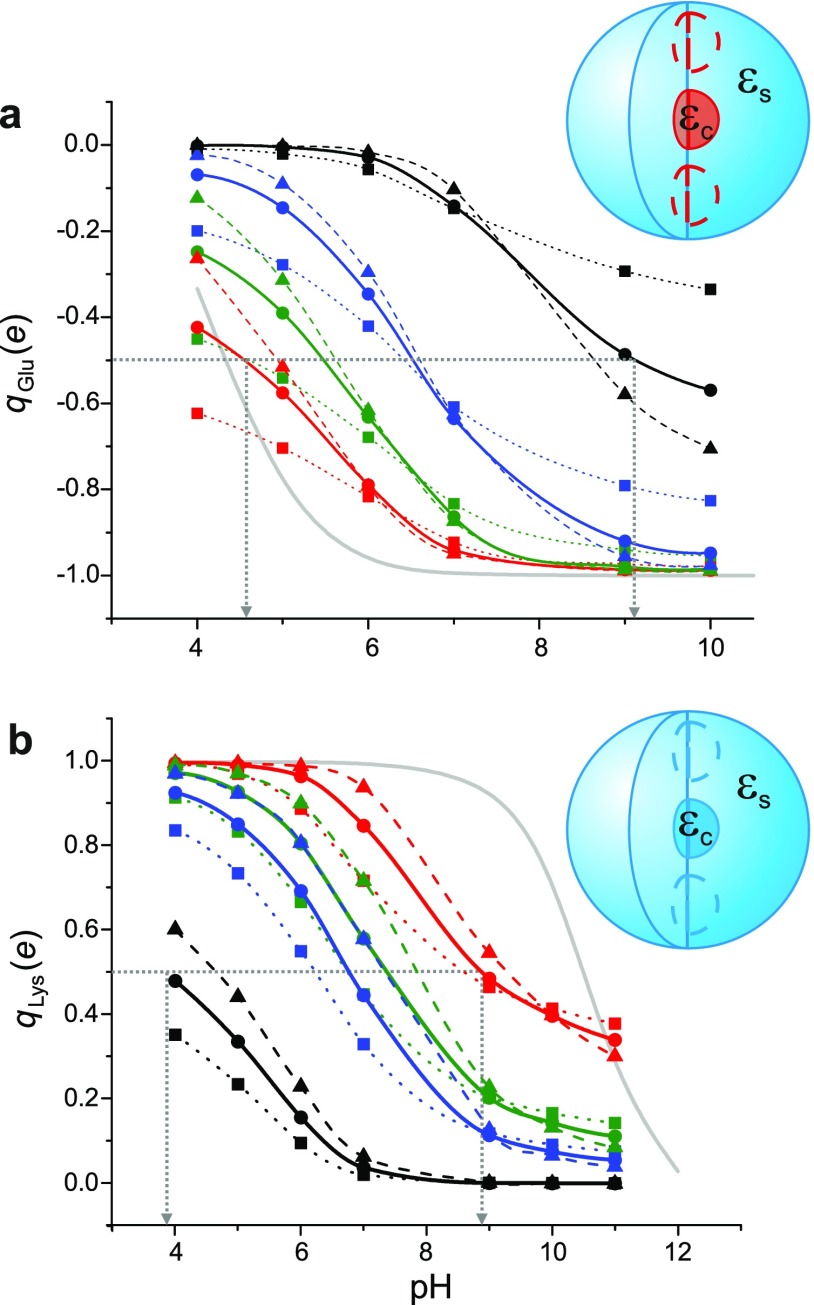
(a) Calculated $\text{p}K_{\text{a}}$ shifts for an interior Glu residue in SNase. The total charge enclosed within the “core” domain housing the Glu residue, $q_{\text{Glu}}$, is plotted as a function of pH, for various values of $\varepsilon_{\text{s}}=25$ (squares), 40 (circles) and 78.5 (triangles), and $\varepsilon_{\text{c}}=$ 25 (red), 20 (green), 15 (blue), 10 (black). The pH at which the charge of the residue is half its fully charged value denotes its $\text{p}K_{\text{a}}$ in the local dielectric environment within the protein. For $\varepsilon_{\text{s}}=40$, the predicted $\text{p}K_{\text{a}}$s ranging from 4.5 to 9.1, and corresponding local dielectric constants $\varepsilon_{\text{c}}=10-25$, agree well with Ref. 23. (b) Calculated $\text{p}K_{\text{a}}$ shifts for an interior Lys residue in SNase. $q_{\text{Lys}}$ is plotted as a function of pH for the same values of $\varepsilon_{\text{s}}$ and $\varepsilon_{\text{c}}$ as in (a). Here, $\text{p}K_{\text{a}}$ values ranging from 3.8 to 8.8 are obtained for local dielectric constants $\varepsilon_{\text{c}}=10-25$, comparable with the results of Ref. 24, using a multiplicative tunable parameter, *p* = 0.25 in the Born solvation energy term in Eq. (3). The grey solid lines denote the charge of an isolated Glu and Lys residue, respectively. All other lines are guides to the eye.

We further compare our model predictions with the results of the same experimental study performed with internal hydrophobic positions replaced by Lysine groups of nominal $\text{p}K_{\text{a}}$ 10.4 (Fig. 7(b)). However in this case, we find that the experimentally measured $\text{p}K_{\text{a}}$ shifts of 4.5 to 9.0 (Ref. 24) are only obtained by introducing a multiplicative tunable parameter, *p*, in the Born energy term in Eq. (3), whose value here is ∼0.25. The need to attenuate the solvation energy contribution in order to capture the experimental measurement in SNase variants with buried lysines strongly suggests that additional descriptive ingredients may be necessary in order to improve the predictive ability of the model.

Currently the model ignores electrostatic contributions such as hydrogen bonds to protein polar atoms and to site-bound water molecules, dipole interactions, structural flexibility and fluctuations, as well as non-electrostatic contributions such as van der Waals interactions to the ionization equilibrium.^43^ Some or all of these contributions could act to offset the effect of the large, energetically unfavorable solvation energy within the protein interior. The fact that the model does not explicitly consider these contributions could point to a physical justification for the introduction of a tunable parameter p<1, or alternatively, the use of $\phi_{0}<0$ in Eq. (3). The effective dielectric constant in our model not only controls the (electrostatic) energy of the sphere but the same parameter also determines the magnitude of the solvation energy. The need to artificially damp the solvation energy contribution in order to obtain agreement with experiment becomes evident in the Lys substitution case because the background charge of SNase is largely positive, creating an *a priori* unfavourable situation for the protonation of a basic group. Nonetheless, our coarse-grained model yields important physical insight into the underlying mechanisms of electrical charging in globular macromolecules.

## V. CONCLUSIONS

The key physical features unique to our integrated model of biomolecular electrostatics in solution are as follows: (1) the transparence of the globular interior to protons in solution, (2) chemical equilibrium of the protons in the interior or on the surface of the molecule with those in the bulk, and (3) an interaction free energy based definition of the renormalized charge. We have tested our model against the following experimental cases: (1) strongly charged polyelectrolytes where only renormalization and almost no charge regulation is expected (DNA), (2) polyelectrolytes where some renormalization and very little regulation is expected (highly charged disordered proteins), (3) globular macromolecules with strong charge regulation and little renormalization, and (4) $\text{p}K_{\text{a}}$ shifts in basic proteins.

We find that our macromolecular electrostatics model successfully captures observations in two very different types of experiment. In escape-time based interaction energy measurements, the model predicts quantitatively accurate values of the effective electrical charge for several biomolecules in the solution phase. In $\text{p}K_{\text{a}}$ shift measurements for globular proteins, it correctly reflects the experimentally observed trends. The success of this framework in capturing experimental measurements on macromolecules in solution, ranging from globular and intrinsically disordered proteins to nucleic acid fragments, given the monovalent salt concentration and pH, suggests broad applicability and the potential to interface with existing platforms for protein electrostatics calculations such as Delphi and APBS.^26,28^

For linear polyelectrolytes, the model predicts that the contribution of charge regulation is small under most conditions of experimental interest. In particular for acidic cylindrical biomolecules with ionizable groups with $\text{p}K_{\text{a}}$s in the range 2-4, such as DNA, charge renormalization rather than regulation plays the dominant role in determining the effective charge. The opposite trend is predicted for globular matter carrying buried ionizable groups. Here in general we expect that charge regulation plays a major role, though a small contribution from renormalization may also be present.

In addition to ignoring ion-ion correlations, and the contribution of molecular charge fluctuations to interaction free energies,^16^ our mean-field PB model currently also neglects ion-specific effects, the finite size of ions, and anisotropy in the dielectric constant of water at interfaces.^44,45^ However it should be possible to account for most of these effects in our model by suitably modifying the PB equation, and incorporating ion-surface interaction potentials and tensorial dielectric functions available from molecular dynamics simulations.^44,45^

Finally, at the level of molecular structure, the model in its current form does not consider the explicit spatial locations of individual charged groups and a local, spatially variable dielectric coefficient within the protein interior. Clearly, further structural detail can be taken into account in future refinements in order to move towards a more exact description of the molecule. Despite its simplicity, the approach provides direct insight into the physical mechanisms underlying macromolecular electrostatics and captures broad experimental trends well. The framework we describe is easy to implement, capable of delivering rapid results of good accuracy, and could find particular relevance in predicting the properties of macromolecules whose crystal structures are not available or are of insufficient quality to reliably apply the atomistic approach.
